# The Hypothalamic-Pituitary-Ovarian Axis, Ovarian Disorders, and Brain Aging

**DOI:** 10.1210/endocr/bqaf137

**Published:** 2025-08-29

**Authors:** Heather Valera, Angela Chen, Kathryn J Grive

**Affiliations:** Molecular Biology, Cell Biology, and Biochemistry Graduate Program, Brown University, Providence, RI 02906, USA; Department of Obstetrics and Gynecology, Program in Women's Oncology, Women and Infants Hospital of Rhode Island, Providence, RI 02905, USA; Department of Obstetrics and Gynecology, Program in Women's Oncology, Women and Infants Hospital of Rhode Island, Providence, RI 02905, USA; Division of Biology and Medicine, Brown University, Providence, RI 02906, USA; Department of Obstetrics and Gynecology, Program in Women's Oncology, Women and Infants Hospital of Rhode Island, Providence, RI 02905, USA; Department of Obstetrics and Gynecology, Warren Alpert Medical School of Brown University, Providence, RI 02905, USA

**Keywords:** sex hormones, gonadotropins, neuroprotection, neurodegeneration, ovarian disorders, endocrine dysfunction

## Abstract

The hypothalamic-pituitary-ovarian (HPO) axis is a complex endocrine feedback mechanism controlling ovulation in female vertebrates. Balance of the HPO axis requires correct secretion of sex steroids from the ovarian follicle to inhibit release of gonadotropins from the pituitary. Several conditions of ovarian dysfunction such as menopause, primary ovarian insufficiency, and polycystic ovary syndrome involve imbalances in the HPO axis, contributing to infertility. Intriguingly, these disorders also share a higher incidence of cognitive and emotional dysregulations, as well as a heightened risk of certain neurodegenerative conditions with age. It is understood that estradiol exerts neuroprotective functions, but gonadotropin signaling is less understood. High concentrations of circulating follicle-stimulating hormone (FSH) and luteinizing hormone (LH) have shown to contribute to neurodegenerative disease states, but are not addressed as part of traditional hormone replacement therapy. To identify the mechanistic connections between ovarian disorders and heightened susceptibility of the brain to pathological aging, a multisystem experimental approach is required, considering each HPO axis player as an individual effector. In this review, we will summarize current knowledge on the effects of estradiol, progesterone, FSH, and LH on neuronal susceptibility to pathology. We will describe ways in which the HPO axis becomes imbalanced during ovarian dysfunction, and how systemic inflammation can become an additional HPO axis effector. Finally, we will recommend solutions to the presented gaps in knowledge, and suggest avenues of future research to pursue development of therapeutics targeting both ovarian and brain health in patients.

Ovarian disorders describe conditions affecting the health of an ovary, often affecting fertility and quality of life. Common symptoms include infertility, vaginal discharge, pelvic discomfort, and weight gain, as well as a host of neurological symptoms including hot flashes, insomnia, mood swings, and brain fog (1-4). Ovarian health can decline due to germline mutations to the oocyte, somatic mutations to oocyte maturation machinery (5), environmental stressors, inflammation, and oxidative stress, among others (6). These conditions are exacerbated by a disruption of the balance of the hypothalamic-pituitary-ovarian (HPO) axis, an endocrine feedback loop in which gonadotropins from the pituitary stimulate sex steroid release in the ovarian follicle. Poor follicular quality often affects the ability of the follicle to respond to gonadotropin cues, causing an imbalance in the HPO axis that contributes both to fertility outcomes and overall symptomatology (7).

A less understood aspect of ovarian disorders is their frequent co-occurrence with neurological conditions, particularly a higher prevalence of dementias later in life, which this review will explore (8). For the purposes of this review, based on terminologies of cited studies, we will refer to people with ovaries as “women”; however, we acknowledge transgender men and nonbinary people also experience these issues. Despite the large proportion of women living with ovarian disorders, the causes of these neurological symptoms remain elusive and understudied. HPO axis imbalance is implicated as a driving factor for neurological phenotypes. A considerable body of literature describes estrogens as neuroprotective, thus their loss would suggest an increased susceptibility to neuronal disorders (9-11). However, direct supplementation of estrogen to patients post menopause has failed to decrease risk of dementias past age 65 years (12). Additionally, elevated serum gonadotropins such as luteinizing hormone (LH) are implicated in the promotion of dementia phenotypes, demonstrating the complexity of the HPO axis in modulating brain health (13). This review focuses on the current body of knowledge surrounding ovarian disorders with regard to the HPO axis, and roles of the HPO axis in susceptibility to dementias. We will then speculate on possible mechanistic connections between ovarian disorder characteristics and resulting neuronal phenotypes, and propose future work in the field to elucidate these mechanisms.

## Ovarian Disorders

### Hypothalamic-Pituitary-Ovarian Axis

Reproductive maturity is established in women on onset of menarche, when the HPO axis becomes active. Growth cues to the brain stimulate a population of neurons in the hypothalamus to secrete gonadotropin-releasing hormone (GnRH) (14). GnRH triggers pituitary release of gonadotropins, namely follicle-stimulating hormone (FSH), to act on ovarian follicles and trigger their growth (15). FSH is required for proper oocyte maturation, largely due to its action on granulosa cells (GCs), the cells that directly surround the oocyte within the follicle. GCs are the primary producers of estradiol, the dominant estrogen during reproductive years. It is a sex steroid responsible for promoting oocyte growth and proliferation of the GCs (14, 16, 17). Successful follicle growth eventually leads to ovulation, an event triggered by pituitary release of another gonadotropin, LH. The ovulated egg travels to the fallopian tube to await fertilization, leaving the GCs to luteinize and form the corpus luteum, a postovulatory structure (18). LH triggers the corpus luteum to secrete progesterone, a sex steroid responsible for maintaining the health of the egg post ovulation. Through endocrine action, estradiol and progesterone both bind to GnRH regulator neurons, as well as gonadotropic pituitary cells to inhibit release of FSH and LH. This negative feedback prevents further estradiol and progesterone production by the GCs, resetting the menstrual cycle (19).

Androgens are the precursor sex steroids to estradiol. Testosterone, an androgen, is the primary sex steroid in males, and is converted to estradiol in the ovary by aromatase. Progesterone is one of the precursors of testosterone production, greatly contributing to androgen synthesis. Although understudied, androgens are implicated in neuroprotective roles, but can also contribute to ovarian disorders when imbalanced (20, 21).

Not in the purview of this review, Insulin-like growth factor (IGF), the somatotropic axis, (14) and the transforming growth factor-β superfamily play roles in fine-tuning the timing and stages of ovulation (22). IGF will be discussed later in the context of insulin resistance contributions to pathology.

### Hypothalamic-Pituitary-Ovarian Axis Disorders in the Ovary

Ovarian disorders are triggered by a wide variety of mechanisms. To achieve proper follicular function, the oocyte must develop correctly in utero, accomplish accurate chromosome segregation, then in the adult mammal respond to growth cues, and secrete prosurvival factors to encourage GC proliferation (23). Steroidogenesis is initiated by gonadotropins and growth factors, and requires sensitivity to gonadotropins to produce sex steroids (14). Secreted steroids must then exert function on brain structures to modulate further release on gonadotropins, or ovulation will not occur (19).

Outlined in Table 1, mutations in genes essential to these processes are linked to several types of ovarian disorders (26, 29, 32, 35). Inflammation can also greatly affect GC-oocyte communication. High oxidative stress conditions in the ovaries of patients with polycystic ovary syndrome (PCOS) can prevent efficient energy production in the GCs, inducing apoptosis and reducing oocyte quality (36, 37). Resulting inflammation from mitochondrial damage and other factors was found to correlate inversely both with steroid biosynthesis rates and embryo quality (38).

**Table 1.** Summary of epidemiology and symptomatology of ovarian disorders

Condition	Prevalence in US	Age of onset, y	Symptomatology	Comorbidities/Complications	HPO axis balance	Genetic linkage examples
Menopause	1.3 million more women per y in US, eventually 100% of women will experience menopause (24).	Mean of 52 in US (25).	Irregular periods, vaginal dryness, hot flashes, night sweats, mood changes, brain fog, insomnia, fatigue (4)	Increased risk of cardiovascular disease, stroke, diabetes, osteoporosis, dementia (4)	High FSH, high LH Low estradiol, low progesterone, low testosterone, low AMH (4).	*PALB2*, *ETAA1*, *HROB*, *BRCA*, *CHEK2* (26)
POI	3.5% globally as of 2021 (27)	Most commonly ages 35-40, globally (1)	Irregular periods before age 40, hot flashes, night sweats, vaginal dryness, mood changes, fatigue, joint pain, loss of focus or memory (1)	Increased risk of dementia, Parkinson disease, heart disease, osteoporosis, hypothyroidism (28).	High FSH, high LH, low estradiol, low progesterone, low AMH, low testosterone (1)	*FMR1*, *PGRMC1*, *AR*, *FOXO4*, *BMP15*, *FS*, *HR*, *STAR*, *CYP17A1*, *BMPR1B*, *GJA4*, *INHA*, *GDF9, ESR1* (29).
PCOS	4%-10% of reproductive-aged women worldwide (3)	Majority of instances ages 20-30 y, globally (3)	Menstrual irregularities, heavy periods, hirsutism, acne, insulin sensitivity, obesity, oily skin, fatigue, mood changes, headaches, insomnia (3)	Increased risk of heart disease, endometrial hyperplasia, uterine cancer, diabetes, dementia (3)	High androgens, high LH, high free testosterone, high AMH. If ovulations are not consistent, low progesterone and high estradiol (30, 31)	*CYP* family, *AR*, *AMH*, *FSHR*, *LH*, *INS*, *IRS-1*, *IRS2* (32)*, THADA* (33)*, DENND1A* (34)

Abbreviations: AMH, antimüllerian hormone; FSH, follicle-stimulating hormone; HPO, hypothalamic-pituitary-ovarian; LH, luteinizing hormone; PCOS, polycystic ovary syndrome; POI, primary ovarian insufficiency.

This is not an exhaustive list of conditions that could be considered ovarian disorders. We have omitted infertility conditions arising from other organs such as hypopituitarism and hyperthyroidism/hypothyroidism (5). The effects of ovarian cancer on the HPO axis are difficult to parse due to noted effects of chemotherapy on cognition not necessarily arising from the cancer itself, so we have omitted these from our list (39).

Endometriosis is an intriguing example of ovarian dysfunction in which the uterine lining forms ectopic lesions on any pelvic organ, including the ovaries. These lesions produce estradiol de novo, increasing circulating estradiol levels. Ovarian endometriosis often induces accelerated follicular atresia due to inflammation, raising FSH levels (2, 40). A Taiwanese study found that patients with endometriosis were 2 times more likely than control individuals to experience a mental disorder, including anxiety, depression, bipolar, sleep disorders, psychotic disorders, and dementia (41). While we can speculate on a potential role for endocrine dyscrasia in the case of brain alterations in ovarian endometriosis, it is difficult to parse this apart from the more established roles of mental illness and chronic pain in increasing susceptibility of the brain to dementias (42). Surprisingly, asymptomatic endometriosis patients were also found to have structural alterations in the brain (43). More research will be required to parse the role of hormonal imbalance in the symptomatology of ovarian endometriosis.

### Menopause

Menopause occurs when loss of oocyte quality from aging accelerates follicular atresia, leaving insufficient follicle counts to produce enough sex steroids for ovulation (24). Healthy women in the United States experience menopause at age 52 years on average (25). The precise timing of menopause is highly hereditary, with several gene mutations linked to onset of symptoms. Intriguingly, most of these genes exhibit roles in DNA damage repair responses, with beneficial variants also increasing risks of certain cancers (26).

Early menopause is defined as a loss of ovarian function occurring between ages 40 and 45 years. Onset of symptoms begins in perimenopause, the transition period into menopause. During this transition, FSH rises, exacerbating follicle maturation (4). Estradiol and progesterone levels become more variable as the follicle pool dwindles, contributing to symptoms. Changes to the brain begin during perimenopause, including decreases in white matter and glucose metabolism in patients (44). Menopause is medically confirmed 12 months after the last period (4). Symptoms lessen during postmenopause, with brain changes stabilizing over time (44).

Menopause increases the risk for comorbidities through several mechanisms. Core body temperature of women post menopause is 0.25 °C cooler on average, causing a 3.25% drop in energy expenditure that would result in 6.7 pounds of weight gain per year in the average woman (45). Increased central adiposity promotes inflammation, contributing to insulin resistance in some patients (46). Menopause increases the risk of dementias and other cognitive symptoms with age, with hormone replacement therapy (HRT) ameliorating this risk only before age 65 years (12). Because of these and other risk factors, age of menopause was found to correlate with mortality and lifespan, with a 2.9% reduction in mortality for every year of later menopause (47, 48).

### Primary Ovarian Insufficiency

Primary ovarian insufficiency (POI) is the absence or early depletion of the ovarian reserve, occurring before age 40 years (1). High FSH, low estradiol, and low antimüllerian hormone (AMH) are all primary characteristics of POI. AMH is produced by GCs and directly correlates with follicle abundance, making it a standard diagnostic tool for POI (49). POI affects around 3.5% of reproductive-aged women globally (27). Genetic factors contribute to around 10% to 15% of cases. X chromosome abnormalities, HPO axis receptors, and steroid producers are among identified mutations (29).

Symptoms arising from POI are extremely similar to those experienced by women in perimenopause. Comorbidities are also similar, including greater risk cardiovascular disease and dementias (28, 29). One study found low-grade chronic inflammation associated with POI, exacerbating aging phenotypes (50). Multiple studies have determined a correlation between age at menopause and lower dementia risk (8). One study in China found that women with a younger age of natural menopause were significantly more likely to experience mild cognitive impairment, as well as receive a dementia diagnosis later in life (51). Patients are often prescribed HRT both of estrogen and progesterone to combat these risks and symptoms (29).

### Polycystic Ovary Syndrome

PCOS is the most common endocrinopathy affecting premenopausal women, with around a 4% to 10% prevalence worldwide (3). PCOS is characterized by hyperandrogenism and high LH (30, 31). Other diagnostic criteria include amenorrhea and inflammatory, fluid-filled cysts present on the ovary. Theca cell steroidogenesis becomes intrinsically activated, aberrantly producing androgens, which modulate FSH signaling in GCs to trigger proliferation. Higher AMH and high follicle counts are reported in PCOS. Impaired timing of ovulation from sex steroid imbalance leads to fluid buildup in follicular structures, creating cysts. Chronic inflammation further propagates androgen production in the follicle (3). Although PCOS is a multifactorial disease, familial studies suggest inheritance may occur in an X-linked polygenic manner (32, 52). Mutations associated with PCOS development include gonadotropin genes, *AMH*, and insulin resistance genes (32).

One study found that by midlife, women with PCOS diagnosis performed worse on fluency tests, and had less white matter integrity than age-matched controls (6). A Canadian study found prevalence of dementia in patients with PCOS to be 2 times higher than in controls, with the median age of diagnosis 19 years younger in patients with PCOS (53). Dietary therapies and oral contraceptives are most commonly used to treat symptoms of PCOS, aiming to reduce free androgen concentrations and improve insulin resistance (32).

## Brain Aging and Neurodegeneration

### Hallmarks of Disease

Neurodegenerative diseases are disorders in which a progressive loss of neurons occurs in the central or peripheral nervous system. As neurons are terminally differentiated cells, maintenance of their health is carefully controlled by numerous mechanisms (54). Disruptions to homeostatic systems are considered hallmarks of neurodegeneration, and were described thoroughly in Wilson et al in 2023 (54). We will briefly describe hallmarks relevant to our topic, including proteostasis and energy metabolism.

Intracellular neuronal health is controlled by autophagy and proteostasis networks. Without recycling of disordered proteins and damaged organelles, aggregates stress intracellular machinery and cytoskeletal transport. Cytoskeletal modulation is particularly important in neurons due to axon length, making efficient transport of nutrients between the synapse and dendrites paramount for neuronal survival and signaling. Aggregates can be deposited in the extracellular space or even passed to other neurons through prion-like spread in extracellular vesicles, causing stress to whole subregions of neurons (54). In Alzheimer disease (AD), protein aggregates of amyloid beta (Aβ) plaques and tau tangles are hallmarks of disease (55).

Control of synaptic signaling is essential for functionality of neuronal networks. Energy supplied by mitochondria allows neurotransmitter release and reuptake. Mitochondrial dysfunction leads to an excess of intracellular calcium, triggering neurotransmitter release to the synaptic cleft that cannot be reabsorbed by the neuron without calcium efflux. This causes an overactivation of neurotransmitter receptors on the receiving neuron. This overactivation, especially by excitatory neurotransmitters like glutamate, can prompt excess calcium influx into neighboring healthy neurons. This phenotype is called “excitotoxicity” and results in the release of reactive oxygen species (ROS), energy depletion, and neuronal death. Proper maintenance of mitochondrial health and neurotransmitter resorption is essential to prevent this feedback (54).

### Neuroinflammation

The brain comprises neurons, glial cells, neuronal stem cells, and blood vessels. While neurons are responsible for information processing, glia perform vital support roles to maintain the health of brain tissue. Microglia are a macrophage-lineage brain-resident immune cell, exerting housekeeping and defensive functions. Overactivation of microglia drives neuroinflammation in several neurodegenerative disorders, causing chronic neuronal stress and eventual cell death. Astrocytes accomplish numerous neuroprotective tasks, including damage responses, neurotransmitter clearance from the synaptic cleft, and maintenance of the blood-brain barrier (BBB). Astrocytes become overactivated in response to excessive stimuli, altering their ability to modulate neurotransmitter recycling and promoting excitotoxicity (54).

The BBB is a vascular barrier composed of endothelial cells, tight junctions, gap junctions, pericytes, and astrocytes. Endothelial cells use specialized transporters to allow the passage of nutrients and oxygen into the brain, while preventing entry of circulating ROS, toxins, and cytokines. Smaller, highly lipophilic molecules such as estradiol can freely cross the BBB, whereas larger, hydrophilic molecules like FSH and LH may require an active transport mechanism to cross (56). Endothelial cells are the most sensitive to damage from inflammatory molecules. Chronic, low-grade inflammation worsens the integrity of endothelial tight junctions over time (57). Astrocytes project endfeet to the endothelial cells, and release numerous factors including IGF1 and apolipoprotein-E to regulate distribution of occludins, claudins, and connexins. Reactive astrocytes downregulate expression of tight junction proteins of the BBB, promoting leakage and exacerbating neuroinflammation. BBB leakage also arises from damage to endothelial cells or neuroinflammation, causing infiltration of immune cells (58) and toxins into the brain, contributing to further neuroinflammation and predisposing the brain to cognitive disorders (54).

### Limbic System

Neurodegeneration can involve disruptions in any or all of the described systems, but some brain regions, such as the limbic system, are especially susceptible to initial damages. Comprising the diencephalon and lower cortex, the limbic system is a highly interconnected region responsible for memory consolidation, emotional and social processing, motivation, and learning (59).

An especially vulnerable limbic structure is the hippocampus, the primary site of adult neurogenesis in the brain. The hippocampus is responsible for learning and memory tasks, specifically memory recall. During healthy aging, the hippocampus experiences one of the steepest declines in volume compared to other brain regions, rivaled only by cerebral white matter (60). The hippocampus is also especially sensitive to hypoxia, making strokes and cardiovascular disease an especially important risk factor for dementia development (61). The hippocampus contains a subpopulation of pyramidal neurons required for learning that have especially high energy demands and an abundance of glucocorticoid receptors, making them extremely sensitive to mitochondrial dysfunction, excitotoxicity, oxygen deficiency, ROS, and toxins (62).

The hypothalamus, also within the limbic system, controls bodily homeostasis through prompting hormone release, controlling sleep-wake cycles, body temperature, and satiety. It is also highly susceptible to damage, with one study finding gene expression signatures with age to be the most changed in the hypothalamus of mice (63). Another study found astrocytes in the hypothalamus were more transcriptionally altered than cortical neurons, potentially hindering their immune-modulating processes (64). In presymptomatic patients with AD, Huntington disease, and amyotrophic lateral sclerosis, hypothalamic alterations related to energy metabolism and caloric intake were altered, suggesting the hypothalamus to be especially vulnerable to early pathologies (65).

The prefrontal cortex is responsible for behavioral regulation, attention, focus, and executive functions. Most publications consider at least the ventromedial frontal cortical regions to be part of the limbic system (66). This area is specifically vulnerable to mitochondrial dysfunction with age. In ovariectomized (OVX) monkeys, morphological changes to mitochondria, as well as increased ROS and oxidative stress, were present in the prefrontal cortex. This effect was rescued by estradiol treatment (67), suggesting a loss of estradiol due to HPO axis dysfunction could affect normal operations. Along with increased susceptibility to disease, the hippocampus, hypothalamus, and limbic system as a whole share high expression of gonadotropin receptors (68), aromatase, and estrogen receptors (69), hinting at possible perturbations to signaling after drastic hormonal change.

## Endocrine Action on Brain Homeostasis

### Sex Steroids

Sex steroids include estrogens, progesterone, and testosterone. Their neurobiological actions are detailed in Table 2. We will focus specifically on the estrogen estradiol, the dominant form of estrogen during reproductive years in both sexes (88, 89). Sex steroids in the brain either enter from circulation or are produced locally by neurons. Neuronally derived estradiol (NDE) has unique effects from circulating estradiol, responding directly to injury, promoting cell survival through brain-derived neurotrophic factor (BDNF) expression, an important neuroprotective factor, and preventing neuroinflammation (70). Aromatase expression is required for NDE production, and is most highly expressed in the limbic system, suggesting damage to the limbic system could lower NDE (69) and promote disease.

**Table 2.** Neuroprotective actions of sex steroids in literature

Sex steroid effector	Neuroprotective activity
NDE	**BDNF induction, learning promotion, prevention of gliosis following injury:** As identified in mouse models lacking aromatase activity in the brain, NDE is important for increasing forebrain synaptic density, efficient synaptic transmission and long-term potentiation, cognitive function, and promoting BDNF expression. NDE also protects against impairment following ischemic brain injury, promotes astrocyte repair mechanisms, and prevents reactive microgliosis. NDE release by neurons and astrocytes is increased following brain injury, likely as a protective mechanism (70).
Estradiol	**BDNF induction**: Estradiol binds an estrogen response element on the BDNF gene, and induces phosphorylation of CREB, both activating and amplifying BDNF to promote neuronal survival, neurogenesis, synapse formation, and synaptic plasticity, especially evident in the hippocampus (10).
Estradiol	**Cell survival, antioxidant activity:** In a study observing the effects of ethanol intoxication on the brain of rats, estradiol was found to activate Sirtuin1 to lower NFκB and TNF-α, preventing neuroinflammation. Estradiol also increased expression of Nrf2 and Ho1 to protect against oxidative stress. Estradiol itself can also scavenge free radicals. Estradiol inhibits neuronal apoptosis by inhibiting caspase 3 and Bax (9), and promoting antiapoptotic effectors B-cell lymphoma (Bcl)-xL and Bcl-w (71). Estrogen receptors are found in neuronal mitochondria, suggesting direct action of estradiol on mitochondria to activate NRF1 and protect against oxidative stress (11).
Estradiol	**Stroke protection:** To prevent onset of ischemic stroke, estradiol can widen blood vessels. Stroke can mechanically damage neurons surrounding the blood vessel, an event that increases risk of dementia onset by 2 to 5 times (72, 73).
Estradiol	**Prevents protein aggregation:** In the context of AD development, estradiol increases PP2a, causing dephosphorylation of tau and stabilizing the cytoskeleton. Estradiol inhibits BACE1, the enzyme that cleaves APP to its pathological form, the antecedent to amyloid plaque formation (69). Estradiol promotes expression of neprilysin, an enzyme that degrades amyloid (74).
Estradiol	**BBB integrity protection:** Estradiol can directly act on cerebral vasculature to suppress inflammatory markers and reduce immune cell adhesion (75). Estradiol promotes survival of endothelial cells, protecting them against inflammation and hypoxia-induced apoptosis (11). Estradiol also acts through ANXA1 to promote BBB integrity. ANXA1 stabilizes cytoskeletal connections in the BBB, prevents circulating immune cell entry, and reduces neuroinflammation. Estradiol mediation of ANXA1 promotes expression of occludin and zona-occludens-1, and reduces expression of ICAM-1 and VCAM-1 to prevent leukocyte invasion (76).
Estradiol	**Prevent insulin resistance:** Estradiol acts through IGF-1 to promote glucose transport and uptake in the brain, with IGF-1 decreasing as estradiol is lost with age, and neuroprotective actions of estradiol hindered without presence of IGF-1 (11).
Estradiol	**Prevents neuroinflammation:** Estradiol acts through ANXA1 to promote a proresolving microglial phenotype, enhancing phagocytosis and anti-inflammatory signatures such as the mannose receptor, CD-206 (77).
Progesterone	**Cell survival:** Progesterone activates MAPK/ERK and PI3K/Akt pathways to promote neuronal survival, and activates BDNF (78).
Progesterone	**Prevent neuroinflammation:** In a multiple sclerosis murine model, progesterone inhibited microgliosis and prevented release of proinflammatory cytokines (79). In a murine model of autoimmune encephalomyelitis, progesterone treatment reduced proinflammatory mediators CD-11b, TNF-α, and lowered activity of nitric oxide synthase, a precursor to oxidative stress (80). Progesterone downregulates inflammasome formation and TNF expression in microglia and induces BDNF release and autophagy in astrocytes (81).
Progesterone	**Promotes BBB integrity:** In a rat ischemia model, progesterone was found to increase tight junction expression through occludin 1 and claudin 5, and reduced inflammation by downregulating metalloproteinases (82).
Testosterone	**Prevents cell death under stress:** Testosterone partially rescued cell death in neuronal cultures experiencing hypoxia and glucose deprivation, dependent on binding to the androgen receptor (83).
Testosterone	**Increases synaptic density through BDNF induction:** Testosterone acts through the androgen receptor to rescue dendritic atrophy in spinal motor neurons after loss of neighboring neuron connections (84). In mice, testosterone can enhance spatial performance, likely due to androgen receptors present in hippocampal pyramidal neurons (85). Testosterone rescued synaptic density in the hippocampus of gonadectomized male mice by inducing expression of BDNF (86).
Testosterone	**Prevent protein aggregation:** Testosterone can downregulate BACE1 to prevent amyloid plaque deposition (87)

Abbreviations: AD, Alzheimer disease; ANXA1, annexin A1; APP, amyloid precursor protein; BACE1, β-secretase; Bax, Bcl-2 associated X-protein; BBB, blood-brain barrier; BDNF, brain-derived neurotrophic factor; cAMP, cyclic adenosine monophosphate; CD, cluster of differentiation; CREB, cAMP response element binding protein; Ho1, heme oxygenase-1; ICAM-1, intercellular adhesion molecule 1; IGF-1, insulin-like growth factor 1; MAPK/ERK, mitogen-activated protein kinase/extracellular signal-regulated kinase; NDE, neuronally derived estradiol; NFκB, nuclear factor-κB; NRF1, nuclear respiratory factor-1; Nrf2, nuclear factor erythroid 2-related factor 2; PI3K/Akt, phosphoinositide 3-kinases/protein kinase B; PP2a, protein phosphatase 2A; TNF-α, tumor necrosis factor-α; VCAM-1, vascular cell adhesion molecule-1.

Research on the neuroprotective effects of estradiol rarely separate effects between NDE and circulating estradiol; however, one can assume that findings from the following research can largely be attributed to the higher concentration of circulating estradiol sourced from the ovaries. Estradiol promotes BDNF signaling induction (10), acts as an antioxidant and promotes cell survival (9, 11), protects against stroke damage (72, 73), prevents insulin resistance (11), and prevents protein aggregation of AD hallmarks Aβ (71) and phosphorylated tau (pTau) tangles (69, 74), effectively protecting neurons from energy loss and excitotoxicity. Intriguingly, female patients with AD have significantly lower levels of estradiol than age-matched controls (90).

Progesterone is also neuroprotective, promoting cell survival through similar mechanisms to estradiol, such as promoting cell survival and reducing neuroinflammation (78). However, progesterone-estradiol combination therapy can cause both positive and negative effects on brain pathology, suggesting the molecules can act both synergistically and antagonistically. In a study testing the effect of estradiol and progesterone on mitochondrial output, estradiol and progesterone could separately rescue mitochondrial function after poisoning, but this effect was lost with coadministration (91). Progesterone can inhibit estrogen receptor expression, as well as prevent estrogen receptors from binding to chromatin to exert their effects. It is unclear in which scenarios this may or may not be occurring, or if another mechanism of action is responsible for this antagonism (78).

Testosterone exhibits some neuroprotective qualities as well, preventing cell death (83), protein aggregation (87), and increasing synaptic density (84-86). However, free testosterone concentration was found to correlate with impaired cognition in women with PCOS, specifically in psychomotor speed and visuospatial learning, suggesting an essential balance of sex steroids to accomplish efficient cognitive function (92).

Regulating neuroinflammation is an essential protective mechanism in the brain. Estradiol and progesterone can both reduce neuroinflammation (77, 79-81 ) and strengthen junctions of the BBB (11, 75, 76, 82). Excessive doses of testosterone in male rats reduced expression of claudin-5 and occludin (93); however, chronic depletion of testosterone also reduced expression of claudin-5, zona occludens-1 (ZO1), and upregulated inflammatory factors tumor necrosis factor (TNF), inducible nitric oxide synthase, and cyclooxygenase-2, suggesting a balance of testosterone concentration is required to maintain BBB integrity (94).

### Gonadotropins

Gonadotropins refer to FSH and LH, which are cyclically released from the pituitary. Their neurobiological actions are detailed in Table 3. Both can become chronically high with loss of sex steroid feedback (15).

**Table 3.** Neurobiological effects of gonadotropins in literature

Gonadotropin effector	Neurobiological activity
FSH	**Increased depressive behavior and neuroinflammation:** Researchers found impaired memory, increased anxiety and loss of pleasure seeking during behavioral testing of male mice administered FSH, and increased TNF-α, IL-6, and activated glia in mouse brains compared to controls. Knockdown of FSHR in the hippocampus reversed these effects (95). In AD mice, treatment with FSH increased GFAP and IBA1, markers for activated astrocytes and microglia, respectively. In OVX mice, these signatures increased as well, with this effect rescued by treatment with an FSH-inhibiting antibody. This effect was also rescued by estradiol treatment (96).
FSH	**Synapse loss:** Synaptic markers synapsin and synaptophysin were decreased following FSH treatment in male mice, with glutamatergic synapse markers GluR1 and VGluT1 decreased as well. Knockdown of FSHR in the hippocampus reversed these effects (95).
FSH	**Increased pathological aggregates:** Experiments in AD mice revealed FSH acts independently of other HPO axis players to bind FSHR in the hippocampus and activate CCAAT/enhancer binding protein β–asparaginyl endopeptidase (C/EBPβ–AEP) to initiate δ-secretase activity, increasing cleavage of APP to its pathological forms. FSH dosing also induced tau phosphorylation and pTau accumulation, as well as amyloid plaque accumulation, and loss of dendritic spines (68).
LH	**Correlation with cognitive deficiency:** In males, LH concentration was correlated with poor memory recall, a mechanism of the hippocampus (97). In male and female patients with AD, LH concentrations were higher than age-matched controls, with LH correlating with cognitive decline, amyloid plaque load, and tau tangles (13).
LH	**Increased pathological aggregates:** Treating neuroblastoma cells with LH increased their secretion of Aβ, due to an increased activity of BACE1, cleaving APP to its pathological form. As Aβ plaque formation can drive hyperphosphorylation of tau, this would further exacerbate AD pathologies (98). Experiments in OVX mice, guinea pigs, and human embryonic stem cells saw increases in APP when treated with LH (99).
LH	**Worsened cognition:** Transgenic mice overexpressing LH had worse cognitive performance on a maze task compared to wild-types; this effect was reversed when LH receptors were nonfunctional (81). When OVX mice treated with estradiol were also treated with an LH homologue, their performance on cognitive and behavioral tests significantly worsened (100).
LH	**Promotion of steroidogenesis:** When treated with LH, rat hippocampal cells and human neuroblastoma cells saw increased pregnenolone productions through upregulation of steroidogenic acute regulatory protein (101). As pregnenolone is a sex steroid precursor and itself neuroprotective, this suggests a nuanced role for LH in disease susceptibility.
FSH + LH	**Reducing BDNF and impairing cognition:** Gonadotropin inhibition via leuprolide acetate in AD mice improved their cognition in behavioral tasks and reduced their Aβ plaque load (102). Similarly, treatment with a GnRH antagonist in OVX rats increased BDNF expression, rescuing cognition (103).
FSH + LH	**BBB modulation:** OVX in mice greatly increased permeability of the BBB, coupled by dysregulation of Cx43, a gap junction. Researchers reported that while OVX increased intensity of Cx43 staining, gonadotropin inhibition lowered Cx43 expression. However, it is unclear what the physiological consequence of this change would be, as Cx43 is vital for gap and tight junction organization, and requires both adequate expression and correct localization to exert proper functionality (104).

Abbreviations: Aβ, amyloid β; AD, Alzheimer disease; APP, amyloid precursor protein; BBB, blood-brain barrier; BDNF, brain-derived neurotrophic factor; Cx43, connexin-43; FSH, follicle-stimulating hormone; FSHR, follicle-stimulating hormone receptor; GFAP, glial fibrillary acidic protein; GluR1, glutamate receptor 1; GnRH, gonadotropin-releasing hormone; HPO, hypothalamic-pituitary-ovarian; IBA1, ionized calcium-binding adaptor molecule 1; IL, interleukin; LH, luteinizing hormone; OVX, ovariectomized; TNF-α, tumor necrosis factor-α; VGluT1, vesicular glutamate transporter 1.

LH exists in two populations—one is released from the pituitary and circulates peripherally, while the other is produced from the hypothalamus and regulates expression levels of LH receptor (LHR) in the brain (105). Peripheral LH levels correlate with AD progression, Aβ load, and cognitive decline in humans (106, 107), but it is unclear if this effect is through a direct action of LH on neurons after crossing the BBB, an indirect action by which high peripheral concentrations of LH suppress brain synthesis of LH, or an entrance of peripheral LH to the brain only after damage to the BBB. Multiple earlier works have found LH to cross the BBB to a limited extent; it is unknown if this action would change under a disease state or state of endocrine dyscrasia (108, 109).

It is clear that high peripheral LH is associated with negative neurological outcomes. In postmenopausal women, high LH correlated with poor cognitive performance (110). Older women with AD had higher levels both of LH and FSH compared to age-matched controls (106). In animal studies, LH administration induces cognitive deficits and increases amyloid deposition (100, 111). Intriguingly, studies directly testing the effect of LH on neuronal cells find mixed results. In neuronal culture, LH increased production of plaques via increased β-secretase (BACE1) activity, but also increased production of pregnenolone, a precursor to the neuroprotective estradiol (98, 101). When administered directly to the mouse brain, one study found LH to impair memory function (112). A separate study found this administration to ameliorate cognitive OVX symptoms (113). Notably, the former study used a much higher dose than the latter. Considering LH can dose-dependently downregulate expression of its receptor, this suggests that benefit or harm could depend greatly on dose. It has been shown that peripheral and brain LH have inverse concentrations naturally (114). Whether or not LH crosses the BBB, high peripheral LH would therefore yield low signaling through LHR, either through suppression of brain LH or through suppression of LHR expression (115). LHR is highly expressed in many disease-sensitive brain regions, including the hippocampus, hypothalamus, cortex, pons, cerebellum, thalamus, and medulla (116), and is an important contributor to memory processes and long-term potentiation through extracellular signal-regulated kinase (ERK) and protein kinase A signaling (117). A reduction in LHR signaling due to high peripheral LH is a plausible theory behind human studies correlating high LH with neurodegenerative indicators. A third possibility is that peripheral LH weakens the BBB, allowing for its own entry to the brain. In OVX mice, FSH and LH inhibition rescued a leaky BBB, indicating one or both of these molecules could allow for its own entry to the brain (104). This would suggest that other factors like toxins or inflammatory cytokines could increase brain concentrations of gonadotropins. Future studies should explore this and other possibilities. For further reading on the subject, see the 2021 review by Mey et al (105).

Many studies correlate FSH concentrations with negative consequences on the brain, but a suggested mechanism has only recently been proposed in the literature. In postmenopausal women, FSH levels correlated with vascular stiffness, increasing stroke risk (118), as well as concentrations of inflammatory cytokines (119). In premenopausal women, FSH levels correlated with depression risk, which itself is a risk factor for dementias (120, 121). FSH receptor (FSHR) brain expression is described in multiple recent studies, with transcripts identified in the mouse cerebellum, olfactory bulb, hippocampus, cerebral cortex, medulla, midbrain and pons, forebrain, thalamus, and hypothalamus (68, 122). Another study validated findings in the yak hypothalamus, pineal gland (123), and mouse hippocampus (124). A further study found FSHR expression in the human cortex, and the mouse cortex and hippocampus (122). Similarly to LH, it is unclear if FSH is able to cross the BBB, if it exerts actions indirectly, or if it contributes to BBB destruction to enter the brain. A recent study using an AD mouse model used a fluorescent tag to observe injected FSH crossing the BBB. The researchers then showed that deleterious effects of FSH were occurring through FSHR binding and subsequent activation of the CCATT/enhancer binding proteins beta and delta (C/EBPβ/δ)-secretase pathway, including deposition of amyloid plaques and tau tangles. They also showed increased gliosis signatures, suggesting FSH modulation of microglia (68). The physical properties of FSH suggest it would have to be actively transported across the BBB, and more research will be necessary to parse how and through what circumstances endothelial transport of FSH would occur. Several studies report FSH can induce inflammatory states in immune cells in peripheral tissues (125, 126), suggesting damage to the BBB through inflammation could be another mode of entry. Overall, more research in the coming years will be required to highlight either a similar or disparate mechanism of FSH action on the brain compared to LH, with current but limited studies hinting at a deleterious and direct action of FSH on sensitive brain regions. For further reading on the topic, see Xue et al (127).

Although neurotypical women are at higher risk of developing dementia than men, men with Down syndrome are more likely to develop the disease than women with Down syndrome. In both cases, the higher risk group experiences elevated concentrations of FSH and LH, suggesting a direct link between gonadotropin concentration and disease risk, regardless of sex (102). Gonadotropin inhibition can induce BDNF expression and rescue cognition (102, 103). More research is required to parse potential regulation of FSH and LH on each other in the brain, with regard to modulation of receptor expression in disease-sensitive brain regions.

## Ovarian Disorders and Brain Pathology

**Figure 1.**
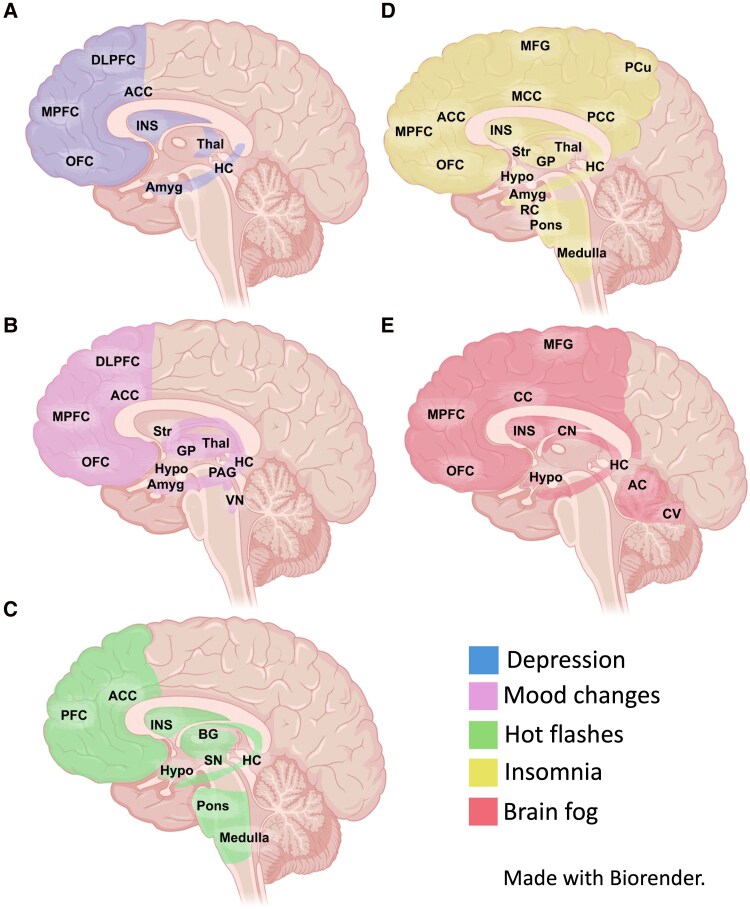
Common symptoms of ovarian disorders share involvement of limbic systems. A, Brain regions involved or altered in depression (130). B, Brain regions involved or altered in mood disorders (131). C, Brain regions involved or altered during hot flashes (132-135). D, Brain regions involved in sleep and altered in insomniac patients (136-139). E, Brain regions involved or altered in conditions using the broad term “*brain fog*” to describe cognitive symptoms (140-143). Abbreviations: AC, anterior cerebellum; ACC, anterior cingulate cortex; Amyg, amygdala; CC, cingulate cortex; CN, caudate nucleus; CV, cerebellar vermis; DLPFC, dorsolateral prefrontal cortex; GP, globus pallidus; HC, hippocampus; Hypo, hypothalamus; INS, insular cortex; MCC, medial cingulate cortex; MFG, middle frontal gyrus; MPFC, medial prefrontal cortex; OFC, orbitofrontal cortex; PAG, periaqueductal gray; PCC, posterior cingulate cortex; PFC, prefrontal cortex; RC, rhinal cortex; SN, substantia nigra; Str, striatum; Thal, thalamus.

This review has described common symptoms and comorbidities of ovarian disorders, and key brain regions susceptible to disease. It is remarkable that many of these symptoms share dysregulation of limbic system structures, a stress-susceptible area heavily populated by gonadotropin receptors (128, 129). Common factors dysregulating activity of these brain structures would heavily implicate HPO axis involvement in symptomatology across the disorders. This is summarized in Fig. 1.

Specific studies highlighting the risk factors discussed in the following sections are displayed in Table 4.

**Table 4.** Neurological risk factors for disease development arising from ovarian conditions, with evidence from literature

Ovarian condition	Risk factor for brain pathology	Studies
Menopause/POI	Lower endogenous estradiol	A study on women with Down syndrome found a correlation between lower endogenous levels of estradiol post-menopause and higher risk of developing AD (144). Estradiol modulates serotonin signaling (145), and low levels of estradiol-target BDNF are correlated with depression onset (146).
Menopause/POI	High LH and FSH blood concentrations	One study in men found that circulating LH concentration directly correlated with circulating levels of Aβ(1-42) (147). A study in women ages 40 to 65 years found blood FSH and LH levels to be positively correlated to Aβ load and negatively correlated with gray matter volume in the frontal cortex (148). In multiple studies, FSH level positively correlated with onset of depression. It is possible that FSH receptors on monocytes signal to produce proinflammatory cytokines, affecting mood (149). Although less is known about LH in the context of mood modulation, one study on rats found that a gonadotropin inhibitor reduced anxious behaviors following ovariectomy (150).
Menopause/POI/PCOS	Insulin resistance.	Evidence of insulin dysregulation was found in hippocampal and cortical sections of patients with AD (151). IGF-1 can promote vasodilation to protect against stroke, promote integrity of the BBB, promote anti-inflammatory glial states, and enhance insulin signaling and glucose uptake by cells (152). Loss of estradiol and IGF-1 and increases in cortisol and insulin resistance predisposing women to stroke onset (55). Insulin resistance also promotes storage of fat in nonadipose tissues and increases appetite, predisposing patients to obesity (153, 154). Insulin resistance promotes vasoconstriction and stroke onset. Insulin itself promotes clearance of amyloid plaques and tau tangles (153, 154). Hyperinsulinemia can cause adipose tissues to become inflamed and release inflammatory cytokines, with this effect amplified in individuals with obesity (155).
Menopause/POI	High cortisol.	Cortisol binds glucocorticoid receptors in the hippocampus and other areas. Chronic activation of the glucocorticoid receptors was found to damage hippocampal neurons by activation of NLRP1 (156). Levels of cortisol in patients with AD correlated with disease severity and progression (157). Cortisol administration to AD mice increased BACE1, Aβ deposition, and tau hyperphosphorylation (158).
Menopause/POI	Chronic inflammation	In patients with POI, serum concentrations of IL-6, IL-8, IL-17, and TNF-α increase with age in patients (159). Similarly, women undergoing menopause had significantly higher serum levels of IL-1β, IL-8, and TNF-α than in fertile women (160).
Menopause	Structural changes.	During the menopause transition, women were found to experience cerebral decreases in white matter, glucose metabolism, and increases in cerebral blood flow and Aβ load. Metabolism shifted to fatty acid utilization, possibly exacerbated by growing insulin resistance. Cognition was not affected during this transition, suggesting compensatory mechanisms occurring as a response to HPO axis dysregulation (44). Mouse models of menopause using OVX mice found a decrease in acetylcholine neuron density, an increase in anxiety, Aβ load, and decrease in locomotion (161).
PCOS	Hyperandrogenism	Androgen treatment to young biological females was found to increase spatial cognition, but worsen verbal fluency (162). Another study correlated high levels of free testosterone in patients with PCOS to worse verbal fluency, verbal memory, and visuospatial memory than controls (92). Several other studies report mixed results, with one finding testosterone correlated with better performance on visuospatial tasks and frontal gyrus connectivity networks, while others report worse performance on spatial visualization, and no effect on cognition. Overall, women with PCOS were found to perform worse on tasks of verbal fluency, verbal learning, verbal memory, visuospatial and executive functions, spatial reasoning, auditory processing, and finger dexterity than controls (163). Treating rats with a nonaromatizable androgen elevated serum levels of TNF-α and IL-1β compared to controls (164). In adipocytes, androgens affect phosphorylation of PKC, downregulating glucose transport and conferring an effect of insulin resistance on metabolism. In turn, hyperinsulinemia stimulates the ovaries to produce more androgens, propagating the cycle (165).

Abbreviations: Aβ, amyloid β; AD, Alzheimer disease; APP, amyloid precursor protein; BBB, blood-brain barrier; BDNF, brain-derived neurotrophic factor; FSH, follicle-stimulating hormone; FSHR, follicle-stimulating hormone receptor; HPO, hypothalamic-pituitary-ovarian; IGF-1, insulin-like growth factor 1; IL, interleukin; LH, luteinizing hormone; NLRP1, NLR family pyrin domain containing 1; OVX, ovariectomized; PCOS, polycystic ovary syndrome; PKC, protein kinase C; POI, primary ovarian insufficiency; TNF-α, tumor necrosis factor-α.

### Menopause and Primary Ovarian Insufficiency

Menopause and POI, or premature menopause, are characterized by high concentrations of serum gonadotropins and low concentrations of sex steroids. Insulin resistance, loss of IGF-1, and heightened cortisol often occur during this transition as well (4). Estradiol and progesterone prevent neuroinflammation and induce neuronal survival and energy homeostasis (144-146), with their loss likely promoting pathology (9-11, 76, 78, 81).

However, HRT post menopause has historically yielded conflicting results in reducing AD risk, with one study concluding that the greatest benefits are yielded if treatment begins in perimenopause, and continues to age 65 years (12). This may suggest that dysregulation of other HPO axis players is also a significant contributor to disease risk. FSH and LH rise considerably during early perimenopause, and may promote protein aggregation (148) and neuroinflammation in animal models (68, 95, 96, 98, 99). Although mechanisms are still being parsed, numerous correlational and human studies and mechanistic animal studies conclude high levels of peripheral LH and FSH, like what occurs during menopause, are detrimental to the health of sensitive brain regions (68, 100, 104, 106, 110, 111, 119).

Additionally, IGF-1 loss, insulin resistance, stroke risk, and cortisol levels all contribute to disease susceptibility. Insulin resistance prevents effective transport of glucose into cells, promoting senescence and hyperglycemia. This causes impaired neuronal glucose metabolism (151), weakened synaptic plasticity, excitotoxicity of glutaminergic neurons, and improper vasodilation of blood vessels (55). Insulin resistance is primarily driven by declines in estradiol, poor sleep and stress, although not all women experience insulin resistance post menopause. Slowing of metabolism leads to increased central adiposity and increased inflammation, contributing to BBB leakage (45, 46). Cortisol also rises in some women during menopause, and can lead to pathological protein aggregation (156-158). IGF-1 loss with age is also attributed to estradiol loss, increasing pathologies of insulin resistance (55, 152).

POI is correlated with oxidative stress and inflammatory aging (159), especially in the case of autoimmune POI, in which the immune system attacks the ovaries. Similarly, women undergoing premature menopause had significantly higher serum cytokines than fertile women (160). This can damage the BBB and promote neuroinflammation.

Besides dementia risk (161), other cognitive effects, such as depression and mood changes, are seen as early as the time of POI diagnosis. The limbic system, which controls emotional responses, is especially sensitive to neuroinflammation and damage (166). High FSH and LH can negatively affect mood (149, 150). Limbic system stress, neuroinflammation, and estradiol loss also contribute to brain fog, insomnia, and hot flashes (167). A combination of high gonadotropins and loss of BBB integrity could potentially exacerbate neuronal symptoms.

### Polycystic Ovary Syndrome

PCOS is characterized most commonly by high androgens, high LH, and high free testosterone. Sometimes patients also experience low progesterone if ovulations are infrequent, and high estrogen due to increased aromatization of androgens. It is speculated that hyperandrogenic conditions desensitize negative feedback mechanisms of sex steroids and hyperexcite GnRH neurons. AMH also activates GnRH firing, perpetuating anovulation while the oocyte population remains high (168). High circulating LH can contribute to protein aggregation and cognitive decline (100, 106, 110, 111), with high estradiol potentially offering a counteracting effect (99, 100, 111).

The effects of hyperandrogenism on cognition are complex, with studies finding both benefits and hindrances on cognition (92, 162). One review summarizes that women with PCOS were found to perform worse on tasks of verbal fluency, verbal learning, verbal memory, visuospatial and executive functions, spatial reasoning, auditory processing, and finger dexterity than controls (163). Some of these effects could be due to hyperandrogenism; however, high LH and psychological stressors could also be contributors.

It is important to note that women with PCOS are more likely to experience depression, anxiety, bipolar disorder, and obsessive-compulsive disorder (169). Physical symptoms like weight gain, chronic pain, and infertility are major contributors to these diagnoses. Intriguingly, increased levels of free testosterone correlated with higher perceived stress in healthy women, suggesting hyperandrogenism could contribute to mental illness (170). Additionally, type 2 diabetic patients were found to have increased cortisol levels compared to healthy controls, suggesting an interplay between insulin resistance and stress (171). Psychiatric disorder diagnosis is associated with an increased risk of dementia development, with disruptions of neurotransmitter networks contributing to excitotoxicity and neuroinflammation (42). Future work should parse the contribution of endocrine imbalance to psychiatric disorder development, as an alternative contributor to dementia onset.

Insulin resistance is a common symptom of PCOS, affecting 65% to 95% of patients (165). Twelve weeks of androgen exposure in rats induced a state of insulin resistance, suggesting hyperandrogenism can induce this symptom (172). Insulin resistance impairs synaptic integrity, promotes stroke onset, and mitochondrial dysfunction of neurons (153, 154). Poor sleep in women with PCOS is directly correlated to insulin resistance, contributing to symptoms of brain fog and insomnia (167).

PCOS is a condition of chronic, low-grade inflammation (155, 164). Increased adipose tissue, insulin dysfunction, and glucose intake all promote further release of cytokines from circulating immune cells (173). This inflammation can weaken the BBB and may induce neuroinflammation.

## Conclusions and Future Directions

**Figure 2.**
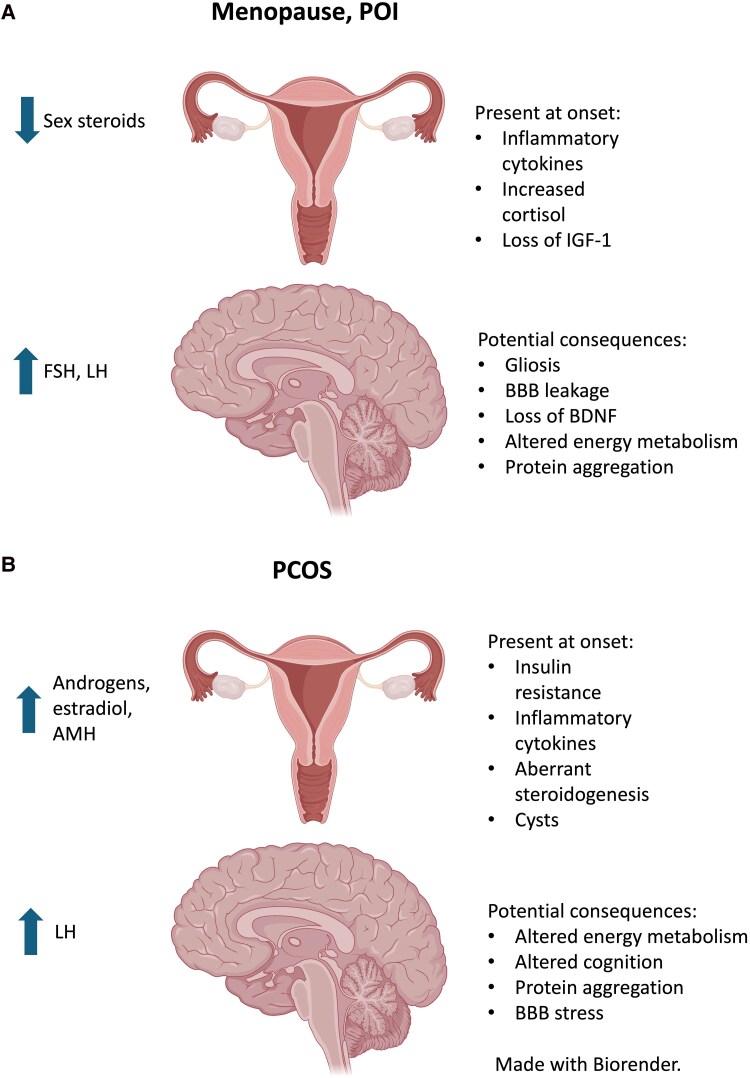
Summary of potential effects of ovarian disorders on brain pathology. A, Menopause and POI comprise inflammatory aging symptoms, with potential consequences of neuroinflammation through BBB disruption, gliosis, and loss of neuroprotection through sex steroid depletion, and pathological aggregation through high gonadotropin concentration. B, PCOS comprises metabolic dyscrasia and hyperandrogenism, with potential consequences of BBB disruption from systemic inflammation, altered cerebral energy metabolism through insulin resistance, and protein aggregation through high peripheral LH.Abbreviations: AMH, antimüllerian hormone; BBB, blood-brain barrier; BDNF, brain-derived neurotrophic factor; FSH, follicle-stimulating hormone; IGF-1, insulin-like growth factor 1; LH, luteinizing hormone; PCOS, polycystic ovary syndrome; POI, primary ovarian insufficiency.

This review highlights the distinct roles of gonadotropins and sex steroids in the brain and reproductive organs, urging a reconsideration of the HPO axis as a network of discrete hormonal effectors with multisystemic effect. Current therapies overlook the effects of ovarian dysfunction on brain health. We suggest an integrative, temporally sensitive, and mechanistically precise approach to understanding and intervening in reproductive aging and its neurological sequelae (Fig. 2).

HRT remains a cornerstone of menopausal care, but evidence suggests neuroprotective benefits diminish after age 65 years (12), demonstrating intervention should begin concurrently with initial hormone dyscrasia, requiring more careful monitoring of endocrine effectors at the suspected time of perimenopause. Additionally, estradiol and progesterone may execute counterproductive actions in the brain when administered together (78, 91). Progesterone administration aids in uterine cancer prevention (174); thus, it could be beneficial to administer hormones more cyclically, minimizing overlap between estrogen and progesterone concurrence. Mechanisms underlying the combined therapy on other organs must be better understood.

To optimize treatment strategies across the spectrum of ovarian dysfunction, future clinical trials should consider levels both of sex steroids and gonadotropins as therapeutic targets. Rebalancing the endocrine environment may be necessary to relieve the range of symptoms experienced in ovarian disorders. For example, gonadotropins rise markedly in perimenopause, making FSH and LH valuable markers for identifying critical windows for intervention. These trials should evaluate cognitive as well as ovarian health outcomes.

A critical next step in HPO axis research is clarifying the mechanistic action of gonadotropins in the aging brain, as questions of BBB access and direct modulation of neurons are inconsistent (113). Future work could prioritize questions of endothelial entry of gonadotropins, as well as the development of specific inhibitors for each gonadotropin, to clarify previous findings using leuprolide acetate to inhibit both gonadotropins simultaneously (102-104). Additionally, existing drugs approved for menopause care that inhibit GnRH pulsatility, such as fezolinetant (brand name VEOZAH, manufactured by Astellas Pharma US Inc.), could potentially be repurposed for improving dementia risk (175, 176). Future trials are needed to assess efficacy of these compounds in improving neuronal outcomes.

Concurrently, BBB disruption in ovarian dysfunction and the potential role of gonadotropins warrant closer study. While animal models of menopause have advanced our understanding of how chronic inflammation compromises BBB integrity (104), comparable models for POI and PCOS remain underdeveloped. Characterizing these models will allow better understanding for ways in which ovarian disorders can contribute to BBB disruption, as well as identifying targetable mechanisms for reinforcing BBB integrity. Therapeutic strategies that modulate local inflammation may also offer long-term benefits, such as using metformin, a diabetes drug, for improving insulin-resistance–derived inflammation in menopause and PCOS (177).

Finally, lifestyle interventions remain a low-risk and potentially high-reward adjunct to pharmacologic approaches. Recent studies have found that increasing dietary intake of fruits, fiber, unsaturated fats, vegetables, and lactobacillus-based probiotics increases beneficial populations of gut microbes, lowering inflammation and improving mood (178-182). Increasing physical activity also lowers vascular markers of inflammation (183) and can improve insulin resistance (184). As part of an integrative therapeutic model, such interventions should be tested in combination with targeted therapies to optimize health-span in individuals with ovarian hormone dysregulation.

In sum, advancing ovarian research requires a reconceptualization of the HPO axis as a network of individual yet interdependent effectors. A multisystem, syncretic experimental approach will be critical to move beyond symptom control and toward a future of personalized endocrine aging management.

## Data Availability

Data sharing is not applicable to this article as no data sets were generated or analyzed during the current study.

## Abbreviations

Aβ: amyloid β
AD: Alzheimer disease
AMH: antimüllerian hormone
ANXA1: annexin A1
APP: amyloid precursor protein
BACE1: β-secretase
Bax: Bcl-2 associated X-protein
BBB: blood-brain barrier
Bcl: B-cell lymphoma
BDNF: brain-derived neurotrophic factor
cAMP: cyclic adenosine monophosphate
C/EBPβ/δ: CCAAT/enhancer binding proteins beta and delta
Cx43: connexin-43
FSH: follicle-stimulating hormone
FSHR: follicle-stimulating hormone receptor
GC: granulosa cell
GFAP: glial fibrillary acidic protein
GluR1: glutamate receptor 1
GnRH: gonadotropin-releasing hormone
Ho1: heme oxygenase-1
HPO: hypothalamic-pituitary-ovarian
HRT: hormone replacement therapy
IBA1: ionized calcium-binding adaptor molecule 1
ICAM-1: intercellular adhesion molecule 1
IGF-1: insulin-like growth factor 1
IL: interleukin
LH: luteinizing hormone
LHR: luteinizing hormone receptor
MAPK/ERK: mitogen-activated protein kinase/extracellular signal-regulated kinase
NDE: neuronally derived estradiol
NFκB: nuclear factor-κB
NLRP1: NLR family pyrin domain containing 1
NRF1: nuclear respiratory factor-1
Nrf2: nuclear factor erythroid 2-related factor 2
OVX: ovariectomized
PCOS: polycystic ovary syndrome
PI3K/AKT: phosphoinositide 3-kinases/protein kinase B
PKC: protein kinase C
POI: primary ovarian insufficiency
PP2a: protein phosphatase 2A
pTau: phosphorylated tau
ROS: reactive oxygen species
TNF-α: tumor necrosis factor-α
VCAM1: vascular cell adhesion molecule-1
VGlut1: vesicular glutamate transporter 1
ZO1: zona occludens-1
